# 

**Published:** 1965-04

**Authors:** 


### Contents

A LIBRARIAN'S VISIT TO AMERICA . *A. E. S. Roberts* 19

CYTOLOGICAL SCREENING FOR PRE-INVASIVE CERVICAL CANCER . . . . . . . .
*J. S. Cornes, Valerie Way, and M. B. Wingate* 23

MUMPS, ARTHRITIS AND PROSTATITIS . *G. Ewart Cree* 27

AORTIC DISEASE: BARBITURATE POISONING . . .
*Clinico-Pathological Conference* 29

LETTER TO THE EDITOR—NSPCC . . . . . . 34

NOTES AND NEWS . . . . . . . . . 35

